# Endoribonuclease L (RNase L) Regulates the Myogenic and Adipogenic Potential of Myogenic Cells

**DOI:** 10.1371/journal.pone.0007563

**Published:** 2009-10-23

**Authors:** Tamim Salehzada, Linda Cambier, Nga Vu Thi, Laurent Manchon, Laëtitia Regnier, Catherine Bisbal

**Affiliations:** 1 INSERM ESPRI 25 “Muscle et Pathologies”, EA4202 Université de Montpellier I, CHU Arnaud de Villeneuve, Montpellier, France; 2 Université de Montpellier II, Place Eugène Bataillon, Montpellier, France; 3 Skuld-Tech, Université Montpellier II, Montpellier, France; McMaster University, Canada

## Abstract

Skeletal muscle maintenance and repair involve several finely coordinated steps in which pluripotent stem cells are activated, proliferate, exit the cell cycle and differentiate. This process is accompanied by activation of hundreds of muscle-specific genes and repression of genes associated with cell proliferation or pluripotency. Mechanisms controlling myogenesis are precisely coordinated and regulated in time to allow the sequence of activation/inactivation of genes expression. Muscular differentiation is the result of the interplay between several processes such as transcriptional induction, transcriptional repression and mRNA stability. mRNA stability is now recognized as an essential mechanism of control of gene expression. For instance, we previously showed that the endoribonuclease L (RNase L) and its inhibitor (RLI) regulates MyoD mRNA stability and consequently muscle differentiation.

We now performed global gene expression analysis by SAGE to identify genes that were down-regulated upon activation of RNase L in C2C12 myogenic cells, a model of satellite cells. We found that RNase L regulates mRNA stability of factors implicated in the control of pluripotency and cell differentiation. Moreover, inappropriate RNase L expression in C2C12 cells led to inhibition of myogenesis and differentiation into adipocytes even when cells were grown in conditions permissive for muscle differentiation. Conversely, over-expression of RLI allowed muscle differentiation of myogenic C2C12 cells even in non permissive conditions.

These findings reveal the central role of RNase L and RLI in controlling gene expression and cell fate during myogenesis. Our data should provide valuable insights into the mechanisms that control muscle stem cell differentiation and into the mechanism of metaplasia observed in aging or muscular dystrophy where adipose infiltration of muscle occurs.

## Introduction

Adult skeletal muscle has an exceptional capacity of regeneration. Indeed, it is capable of responding to physiological stimuli for routine maintenance or after a severe injury by complete repair of the tissue. This feature is due to the presence of satellite cells, a quiescent population of resident stem cells that are located beneath the basal lamina and which can generate large quantities of differentiated muscle cells [1]–[3]. Furthermore, due to their capacity to regenerate damaged muscle, satellite cells have been considered as candidates for cell-based therapies to treat muscular dystrophies or other muscular diseases characterized by loss of muscle cells [4].

However, perturbations in muscle cell differentiation can be observed *in vivo* in physiological situations, such as aging [5], or in pathological conditions. For instance, adipose infiltration occurs during type 2 diabetes and obesity, muscle denervation [6]–[8] and in some muscle diseases, such as muscular dystrophy [9] and mitochondrial myopathy [10]. Several works have demonstrated that myogenic cells express some adipogenic markers and that, following specific stimulation, they can differentiate, at least *in vitro*, into adipocytes [11], [12]. Therefore, myogenic cells can potentially differentiate both into myoblasts and adipocytes and in some conditions a change in differentiation program could take place.

Deciphering the molecular mechanisms that control the proliferation and the possible alternative differentiation pathways of myogenic cells is, therefore, central to understanding their function and also is important in view of the use of satellite cells for therapy. The particular anatomical localization of satellite cells allows a combination of signals from the muscle fiber itself, the circulatory system and the extracellular matrix (ECM) to govern their quiescence, activation and proliferation [13].

Interferon (IFN) is one of the cytokines present in the satellite cell environment. It is produced by several cell types and also by muscle cells during skeletal muscle regeneration [14]. The involvement of IFN and IFN-induced proteins in the regulation of muscle cell growth and differentiation has been long established [15]–[19]. We have previously shown that the IFN-induced 2-5A/endoribonuclease L (RNase L) and RNase L Inhibitor (RLI), its inhibitor, play a role in myoblast differentiation by regulating the stability of MyoD mRNA [20]. IFN induces the expression of 2-5A synthetase which, upon activation by double stranded RNA, converts ATP into an unusual series of oligomers known as 2-5A [21]. 2-5A activate RNase L, a latent endoribonuclease, which inhibits protein synthesis by cleaving mRNAs at the 3′ side of UpNp sequences.

Our previous work showed that RNase L activity is normally and transiently induced during myoblast proliferation reaching a maximum when cells are confluent. Concomitantly, 2-5A synthetase also is induced allowing RNase L activation [15], [22]. RNase L activity is then inhibited by induction of RLI during induction of muscle-specific genes and cell fusion [20].

In the present work, we wanted to extend our understanding of the role of RNase L during muscle differentiation. By comparing global gene expression in C2C12 cells over-expressing RNase L and in control cells using SAGE (Serial Analysis of Gene Expression) [23] we identified different transcripts that are down-regulated by RNase L. Many of these mRNAs encode key factors implicated in the control of pluripotency, commitment and differentiation of stem cells. Some of these factors, like Aebp1 or Chop-10/Ddit3, are implicated in the choice between alternative differentiation pathways. Moreover, we show that increasing RNase L expression in C2C12 myogenic cells at an inappropriate time, such as early during the multipotency phase, favors their adipogenic potential rather than their myogenic potential. Conversely, RNase L inhibition by increasing RLI expression leads to muscle differentiation even in non-permissive culture medium.

## Materials and Methods

### Cell culture

For conditional expression of human RNase L, the LacSwitch II inducible mammalian expression system (Stratagene) was used. The C2C12 clone in which RNase L is conditionally expressed (C2-RNase L) was previously described [20]. For induction of RNase L, C2-RNase L cells were cultured in growth medium (GM) [DMEM (Cambrex) and 10% (V/V) fetal calf serum (FCS) (Dutcher)] and at 80% confluence were treated with IPTG (Isopropyl-β-D-thiogalactopyranoside) as indicated in the figure legends.

C2-RLI cells, which over-express RLI, were previously described [20]. These cells were routinely maintained in GM at low cell density (300 cells/cm^2^) [20].

### Differentiation of C2-RNase L and C2-RLI cells

For differentiation experiments, C2-RNase L cells were plated at high density (10^4^ cells/cm^2^) in GM. When cells reached confluence, they were switched to differentiation medium. To assess muscle differentiation, cells were grown in muscle differentiating medium (MDM) (DMEM supplemented with 2% (V/V) FCS) for 6 days. At different time points, as indicated in the figure legends, cells were washed with PBS, fixed in 10% (V/V) formalin for 10 min and permeabilized with PBS-Triton X100 0.5% (V/V) at room temperature for 5 minutes. After blocking with 10% (W/V) FCS in PBS, cells were incubated with a mouse monoclonal anti-Troponin T antibody (1/1000; Sigma) or a rabbit monoclonal anti-Fabp4/aP2 (1/100; Cell Signaling) at room temperature for 1 h. Cells were then washed and incubated with a donkey anti-mouse secondary antibody conjugated to TRITC or FITC (Santa-Cruz) or a donkey anti-rabbit secondary antibody conjugated to TRITC (Santa-Cruz) at room temperature for 1 h. For adipocyte differentiation, cells were cultured in adipocyte differentiating medium (ADM) [DMEM-10% (V/V) FCS, 5 µg/ml Bovine insulin (Sigma) and 1 µM dexamethazone (Sigma)] for 6 days. Lipid cells were visualized by staining with Oil-red-O [24]. At different time points (see figure legends) cells were washed twice with PBS, fixed with 10% (V/V) formalin for 15 min, and then immunostained with an anti Fabp4/aP2 or a rabbit anti-Perilipin antibody (1/200; Cell signaling) as described above or stained with 0.3% Oil-red-O (Sigma) in isopropyl alcohol: distilled water (60∶40) at room temperature for 60 min and then washed with water to remove unbound dye. Lipid accumulation was quantified by spectrophotometric analysis at 540 nm after eluting the Oil-red-O retained in the cells with isopropanol [25].

For differentiation experiments, C2-RLI cells were plated at high density (10^4^ cells/cm^2^) in GM and, at confluence, they were switched to ADM as described above. Lipid and muscle cell phenotypes were characterized as described above.

### Analysis of RNase L over-expression

RNase L expression was followed with the 2-5A radio-covalent or radio-binding assay [26]. C2-RNase L cells, C2C12 cells or C2-RLI cells were collected, resuspended in 2 volumes of hypotonic buffer (20 mM HEPES, pH 7.5; 10 mM potassium acetate; 1.5 mM Magnesium acetate; 1% [vol/vol] NP-40; 1 mM phenylmethylsulfonyl fluoride; 10 µg of aprotinin per ml; 150 µg of leupeptin per ml), disrupted in a Dounce homogenizer, and centrifuged at 10,000×*g* (S10). The protein concentration in the supernatant (S10) was determined by spectrophotometry [27]. For radiobinding, cell extracts (600 µg of protein) were incubated with 20,000 cpm of 2-5A_4_-3′-[^32^P]pCp (2-5ApCp; 3,000 Ci/mmol) on ice for 15 min as previously described [28]. Radiolabeled RNase L was then precipitated at −20°C for 5 min by using 300 µl of polyethylene glycol 6000 (25% [wt/vol]) after addition of 150 µl of bovine serum as a carrier. After centrifugation (10,000×*g*, 10 min), the radioactivity of the pellet containing the 2-5ApCp bound to RNase L was measured. Experiments were done in triplicate and the standard deviation is indicated on the plots. For the radio-covalent assay, cell extracts (200 µg of protein) were incubated with the oxidized 2-5A_4_-3′-[^32^P]pCp probe [28]. Labeled proteins were separated by sodium dodecyl sulfate-polyacrylamide gel electrophoresis (SDS-PAGE) [29] and were visualized by autoradiography. Protein bands were quantified by image analysis with the Intelligent Quantifier program (Bio Image Systems Corp.). Experiments were done in triplicate and the standard deviations are indicated on the plots.

### RLI quantification

C2-RLI cells were collected and proteins extracted as described above. Proteins (100 µg) were fractionated by SDS-PAGE and transferred electrophoretically to nitrocellulose membranes. Membranes were then blocked with 5% (W/V) skimmed milk in phosphate-buffered saline (PBS; 140 mM NaCl, 2 mM KCl, 8 mM Na_2_HPO_4_, 1.5 mM KH_2_PO_4_ [pH 7.4]) for 30 min and incubated with a rabbit polyclonal anti-RLI antibody (1/500) (Novus Biological) in the same buffer at 4°C overnight. Membranes were washed in PBS supplemented with 0.05% (V/V) Tween 20 and incubated at room temperature with a donkey anti-rabbit IgG conjugated to horseradish peroxidase (Amersham) for 1 h. Specific proteins were visualized using a chemiluminescence kit (Pierce). Films were scanned, and protein bands quantified by image analysis with the Image J software. Experiments were done in triplicate and the standard deviation is indicated on the plots.

### rRNA cleavage

C2-RNase L cells were harvested during the proliferative or multipotency phase after RNase L induction with 5 mM IPTG for 6 hours. Cells were resuspended in 1 volume of buffer (5 mMTris-HCl pH 7.6; 1.25% (V/V) glycerol; 20 mM KCl; 1.25 mM magnesium acetate), vortexed for 1 min and kept on ice for 10 min. The cell buffer suspension was passed through a 1-ml tuberculin syringe and centrifuged at 15,000×g for 2 min. Cell extracts (100 µg of total proteins) were incubated at 30°C in the presence or absence of 2-5A_4_ (1 µM, final concentration) for 1 h. After extraction with TRIzol (Invitrogen) following the manufacturer's instructions and agorose gel analysis, total RNA bands were visualized under UV light [30].

### Construction of SAGE libraries

C2-RNase L cells were seeded at high density in GM. At 80% confluence cells were treated or not with 5 mM IPTG for 6 hours. Then, cells were harvested and RNAs extracted with TRIzol (Invitrogen) following the manufacturer's instructions. RNA quality was checked by analysis of total RNA on 1% agarose gel and visualization of ribosomal bands with ethidium bromide. 80 µg of total RNA from C2-RNase L cells treated with IPTG or not were used to construct the two SAGE libraries with the I-Sage kit (Invitrogen) based on the technique developed by Velculescu [23]. After cloning the concatemers in pZero, the libraries were electroporated into TOP10 *E. Coli* competent cells. Transformants were screened by PCR amplification and inserts >400 nucleotides (28 tags) were sequenced.

### Analysis of SAGE libraries

SAGE libraries were analyzed with the CplusTag and Preditag software programs developed by Skuld-Tech (Montpellier, France) [31]. CplusTag was written in C and implemented on a UNIX operating workstation for automatic tag detection and counting. This program provided criteria for assessing the quality of the SAGE libraries (length distribution of ditags, frequency of replicate ditags, and detection of linkers). Ditags with less than 20 bp were discarded and repeated ditags were not taken into account for counting the tag.

#### Tag prediction

Mm.seq.uniq and Mm.data files were downloaded from the UniGene FTP site at NCBI (ftp://ncbi.nlm.nih.gov/repository/UniGene). A table was constructed by extracting virtual tags from the representative sequences associated with each UniGene cluster in an Mm.seq.uniq file, then by parsing attributes associated to each cluster in the Mm.data file. Several UniGene qualifiers were used directly as column titles, providing the framework for the main table of the database. An URL was created for each putative tag, establishing a link with the UniGene web site. Other fields were created for recording additional data, including the presence or not of a polyadenylation signal in the reference sequence, the distance between the anchoring enzyme site and the end of the sequence, and criteria for evaluating risks of multiple matches. Full-length cDNAs were distinguished from ESTs by checking that their sequence was provided by large-scale sequencing programs (for example, DKFZ). Data were retrieved at EBI (ftp://ftp.ensembl.org) for the subset of well-annotated genes (ensembl.cdna). Microsoft Access functions were used for tag-to-gene assignment and subsequent data management. A query using the tag sequence as the primary key allowed us to match experimental sequences (CplusTag), virtual sequences (Preditag) and selected annotations, thus generating a table of results. Sequencing of these two libraries allowed the identification of 8367 (Control SAGE) and 7014 (RNase L SAGE) mRNAs and ESTs which represent 3757 different mRNAs and ESTs expressed one or more times. A list of the genes, which were >3 fold down-regulated upon induction of RNase L, is provided in supplementary Table S1.

### Semi-quantitative RT-PCR amplification

Total RNAs were isolated using TRIzol (Invitrogen). To avoid genomic DNA contamination and amplification during PCR analysis, RNAs were treated with RNase-free DNase (Euromedex) before reverse transcription. Complementary DNAs (cDNAs) were generated by RT with oligo-(dT) primers and the Superscript II enzyme (Invitrogen). Briefly, 3 µg of total RNA was denatured at 70°C and then reverse-transcribed with Superscript II at 42°C for 50 min. Gene sequences for primer design were obtained from the NCBI Reference Sequences database. Primers were chosen using the Primer3 Software except for the forward and reverse EEF1α primers which were the controls included in the I-Sage kit (Invitrogen). When possible, the forward and reverse primers for GAPDH, Aebp1, Ddit3, Stat3, Vimentin, Calponin 2, Nischarin, RIL, HDAC5, Septin 7, PPARGγ2, MyoD, Pax7, Myogenin, RLI, C/EBPa, aP2, OAS1 (2-5A-synthetase 1) were selected from different exons sequences. Primer sequences are provided in supplementary Table S2. PCR amplification (30 cycles) was performed in a total volume of 25 µl and the amount of each cDNA was adjusted for each primer pair to be in the linear range of amplification and to give the same quantity of amplified complementary DNA as with the EEF1α primers. PCR products were run in 1.2% agarose gels, stained with ethidium bromide and bands quantified with the Image J program. Experiments were done in triplicate and the standard deviations are indicated on the plots.

### mRNA stability

C2-RNase L cells were plated at high density in GM, and, at 80% confluence, RNase L was induced by IPTG (2 mM during 6 h). Then cells were treated with Actinomycin D (5 µg/ml) for 0, 1, 2, 4 or 6 hours and RNAs were extracted and analyzed by semi-quantitative PCR amplification as described above. Cells were treated with only 2 mM IPTG to avoid excessive mRNA degradation by RNase L and to allow mRNA quantification after Actinomycin D treatment. After agarose gel electrophoresis, gels were scanned and bands quantified with Image J. Experiments were done in triplicate and the standard deviations are indicated on the plots.

## Results

### Transcriptome analysis

C2C12 cells are myogenic cells that can be used as a model system to study the early stage of myoblast [32] and adipocyte [33] differentiation, depending on the cell culture conditions. We have previously shown that in C2C12 cells, in which an inducible expression system allows conditional over-expression of RNase L (C2-RNase L cells) by addition of Isopropyl-β-D-thiogalactopyranoside (IPTG) in the culture medium [20], MyoD was down-regulated and myogenesis inhibited. To extend our knowledge about the role of this mRNA degradation pathway in the commitment of C2C12 cells during differentiation and to identify mRNAs regulated by RNase L, we have performed global gene expression analysis using the SAGE (Serial Analysis of Gene Expression) technology [23] in control C2-RNase L cells (not treated with IPTG) and C2-RNase L cells over-expressing RNase L (treated with IPTG).

**Figure 1:**
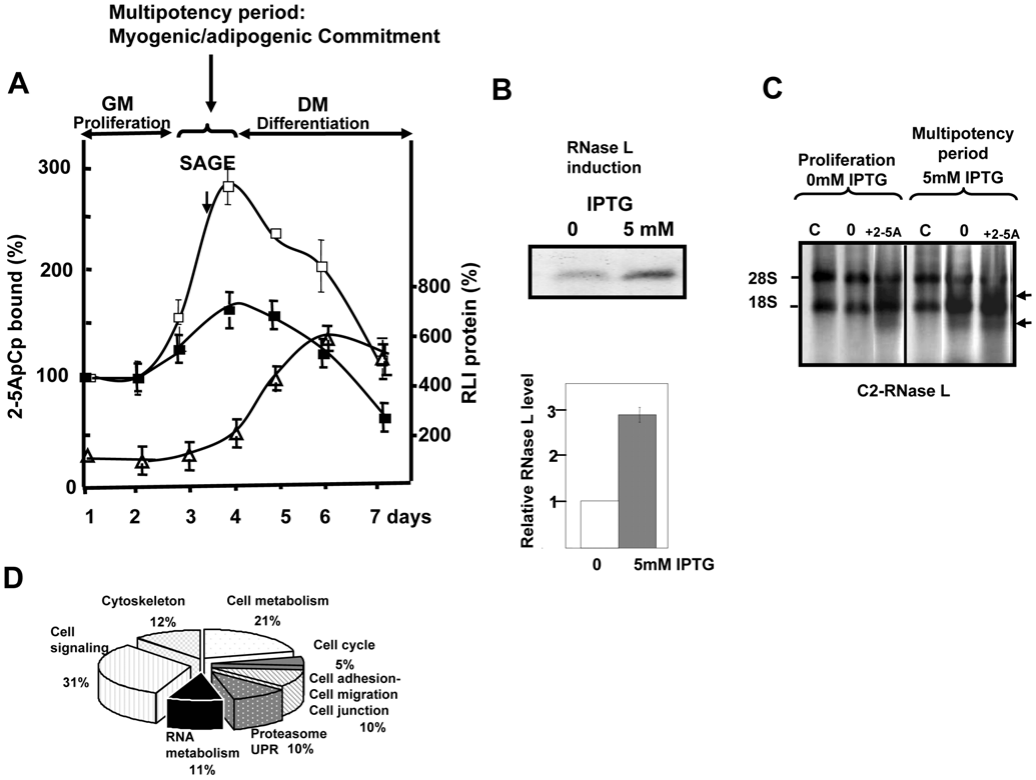
Scheme of SAGE analysis. A: RNase L expression during myotube differentiation. C2-RNase L cells were plated at high density in GM (day 1). At day 3 (80% confluence), C2-RNase L cells were treated (-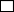-) or not (-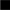-) with 5 mM IPTG for 6 hours and shifted to MDM at confluence (day 4). At the indicated time points, cells were harvested. To quantify RNase L, proteins (100 µg) were incubated with radio-labeled 2-5A_4_-3′-[^32^P]pCp (2-5ApCp) in a radiocovalent assay; 100% corresponds to the amount of 2-5ApCp bound to RNase L in proliferating myoblasts at day 1. Error bars refer to the standard deviation obtained in two independent experiments. Gels were scanned and protein bands quantified by image analysis with the Image J program. Standard deviations are indicated on the plots. RLI expression was assessed in protein samples (100 µg) using an RLI-specific antiserum. Gels were scanned, and protein bands quantified by image analysis with Image J. The experiments were done in triplicate and standard deviations are indicated on the plots ( -Δ- ). B: Induction of RNase L. For SAGE experiments, C2-RNase L cells were treated, or not, with 5 mM IPTG for 6 hours at day 3 (time indicated with an arrow in panel A). Cells were then harvested and analyzed for RNase L 2-5A binding activity with the 2-5A radiocovalent binding assay. Proteins were separated in 10% polyacrylamide gels. A representative autoradiography and a densitometry of the gel are shown. The amount of 2-5A binding activity in C2-RNase L cells in the absence of IPTG was set to 1. Error bars refer to the standard deviation obtained in three independent experiments. C: Specific RNase L rRNAs cleavage activity. C2-RNase L cells were plated at high density in GM (day 1). At day 3, cells were treated with 5 mM IPTG for 6 hours. Cells were harvested at day 2 (proliferation) and day 4 (at confluence and after RNase L induction) and cell extracts (100 µg proteins) were incubated or not (C) at 30°C with 1 µM 2-5A_4_ for 60 min. After extraction, rRNAs were analyzed on 0.8% (WT/V) agarose gels. Intact 28S and 18S bands (rRNA from control, untreated cells) are indicated at the left of the gel. Major rRNA degradation products are indicated by arrows at the right side of the gel. D: Classification by function of the down-regulated genes.

The nuclease activity of RNase L strictly depends on its activation by 2-5A, therefore IPTG treatment should be performed when 2-5A synthetases are present. Others and our previous work showed that RNase L activity and 2-5A synthetases (OAS1, 40/46 kDa and OAS2, 69/71 kDa) [21] are normally transiently induced at the end of the proliferative phase of myoblasts, during the multipotency period, and reach a maximum when cells are confluent (Figure 1A) [15], [20], [22]. For this reason, we chose to induce RNase L expression in 80% confluent C2-RNase L cells, i.e., when they can still be committed to different differentiation pathways (Figure 1A). RNase L induction was monitored by binding to 2-5ApCp (Figure 1A and 1B) and quantification of the western blots indicated an increase in the concentration of RNase L compared to control C2-RNase L cells not treated with IPTG. RNase L induction in the presence of 2-5A synthetases normally leads to activation of RNase L, as also shown in Figure 1C. RNase L activity was monitored *in vitro* by studying the specific cleavage pattern of rRNA [30]. We could observe rRNA cleavage even without addition of 2-5A when RNase L was induced at a time when 2-5A-synthetases are present and active in the cells (i.e., in 80% confluent cells) (Figure 1C).

On the basis of these preliminary results, cells were harvested for RNA extraction 6 h after addition of 5 mM IPTG, which represented the shortest incubation time needed to obtain a good induction of RNase L without stimulation of apoptosis due to over-activation of RNase L [34], [35]. Then two SAGE libraries were constructed as previously described [23], [31]. To identify RNase L targets we focused on transcripts that were down-regulated upon RNase L induction (Supplemental Table S1). Despite its weak sequence specificity, RNase L induction did not lead to global RNA decrease, as only 4% (166/3757) of the identified transcripts were down-regulated more than 3 folds. Functional classification of these mRNAs indicated that they encoded for proteins involved in different cellular processes and pathways (Figure 1D) which are important also for the regulation of muscle differentiation. For instance, among the transcripts involved in cytoskeleton organization, cell shape and differentiation, we identified Pdlim4/RIL, a gene down-regulated in H-ras transformed cells [36], which forms with ALP, Elfin and Mystique the ALP subfamily of PDZ-LIM proteins [37] and is a regulator of actin stress fiber turnover [38]. Septin 7, a GTP/GDP-binding protein that assemble into filamentous cytoskeletal polymers, is involved in the organization of several structures such as the actin stress fibers, cell shape and signal transduction by sequestering key signaling molecules [39], [40]. Zyxin was the first focal adhesion protein shown to shuttle between focal adhesion site and nucleus. In the cytoplasm, Zyxin influences actin assembly and organization as well as cell motility [41]. Nischarin alters actin filament organization and inhibits cell motility [42]. Vimentin and Nestin are components of the intermediate filaments and are expressed in replicating myoblasts and early myotubes [43], [44]. Finally IqSec1 (Brag2/GEP100), a guanine exchange factor of ADP ribosylation factor 6, plays a central role in myoblast fusion and myotube maturation [45].

Another group of transcripts strongly down-regulated by induction of RNase L encoded for factors which are critical for maintaining pluripotency in stem cells or for cell fate commitment. One of these factors, Stat3, plays an essential role in myoblast proliferation and actively prevents their premature differentiation [46], [47]. Stat3 maintains silencing of differentiation-associated genes also in self renewing ES cells [48]. The Wnt receptor Fzd7 is also critical for maintaining pluripotency and self renewal of ES cells [49], [50]. Chop-10/Ddit3, a member of the C/EBP family of transcription factors, plays a role in cell survival and differentiation, inhibits adipogenesis and induces osteoblastogenesis [51], [52]. Aebp1 is a transcriptional repressor that is down-regulated during adipogenesis [53]. Hdac5, a chromatin-remodeling enzyme, interacts with MEF2 proteins resulting in repression of MEF2-dependent genes [54], [55]. The *H19* locus produces a 2.5-kb non-coding, spliced and polyadenylated RNA of unknown function and regulated by imprinting [56]. We have previously shown that *H19* expression is up-regulated exclusively by stabilization of its RNA during muscle cell differentiation [57]. Recent work reported that such non coding RNAs have functions in diverse processes from embryonic stem cell pluripotency to cell proliferation [58]

### RNase L directly regulates master genes that control pluripotency and cell fate

**Figure 2:**
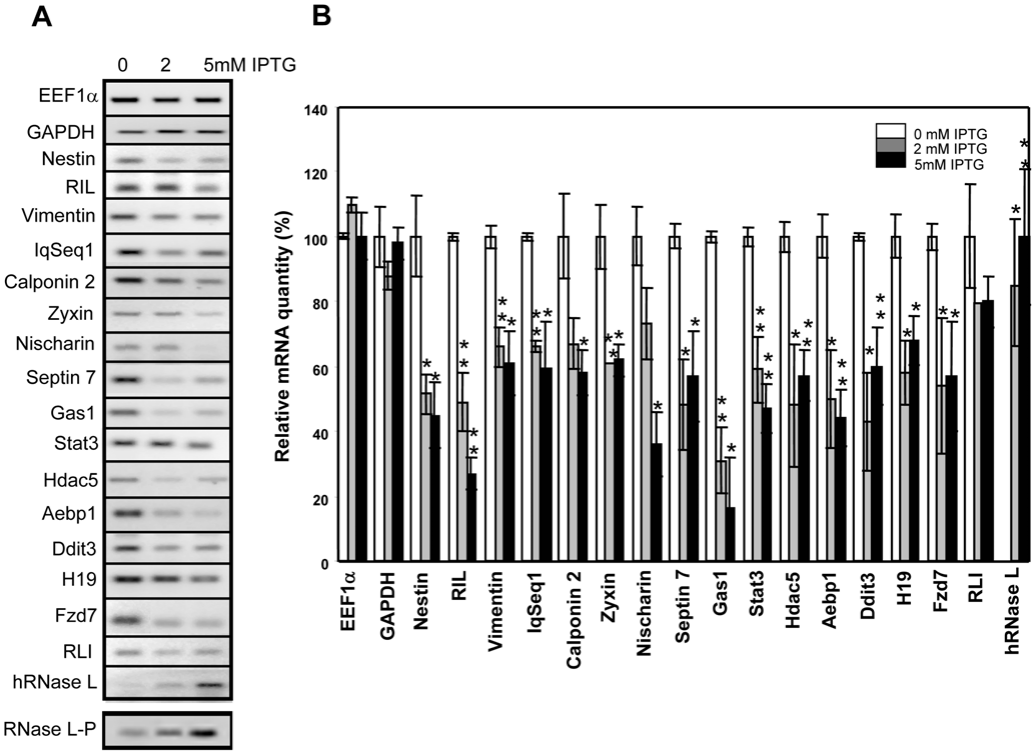
Down-regulation of the identified mRNAs depends on the RNase L level. A: C2-RNase L cells were treated or not with 2 or 5 mM of IPTG for 6 h. mRNAs were quantified by RT-PCR amplification using specific primers (Supplementary Table S2). PCR products were analyzed on 1.2% agarose gels. Photographs of the gels are shown. Induction of RNase L was also analyzed at the protein level as in Figure 1B. An autoradiography of the gel is shown (RNase L-P). B: Densitometry of the gels shown in panel A. The level of the different mRNAs in untreated cells was set at 100%. Quantification of EEF1α mRNAs was used as a control of each semi-quantitative RT-PCR experiment. Error bars refer to the standard deviation obtained in three independent experiments. (*) P<0.05 and (**) P<0.01 compared to control, untreated cells.

To experimentally validate the SAGE data, we analyzed by semi-quantitative RT-PCR amplification the expression level of some of the identified transcripts, which could be of relevance for muscle differentiation, and of two negative controls (EEF1 α and Gapdh) upon induction of different RNase L concentration by treating, or not, C2-RNase L cells with 2 or 5 mM IPTG for 6 h (Figure 2). This analysis confirmed that most of these mRNAs were down-regulated upon induction of RNase L and the intensity of this effect was proportional to the amount of RNase L induced. Induction of human RNase L expression both at the mRNA and protein level was proportional to the IPTG concentration used (Figure 2A).

**Figure 3:**
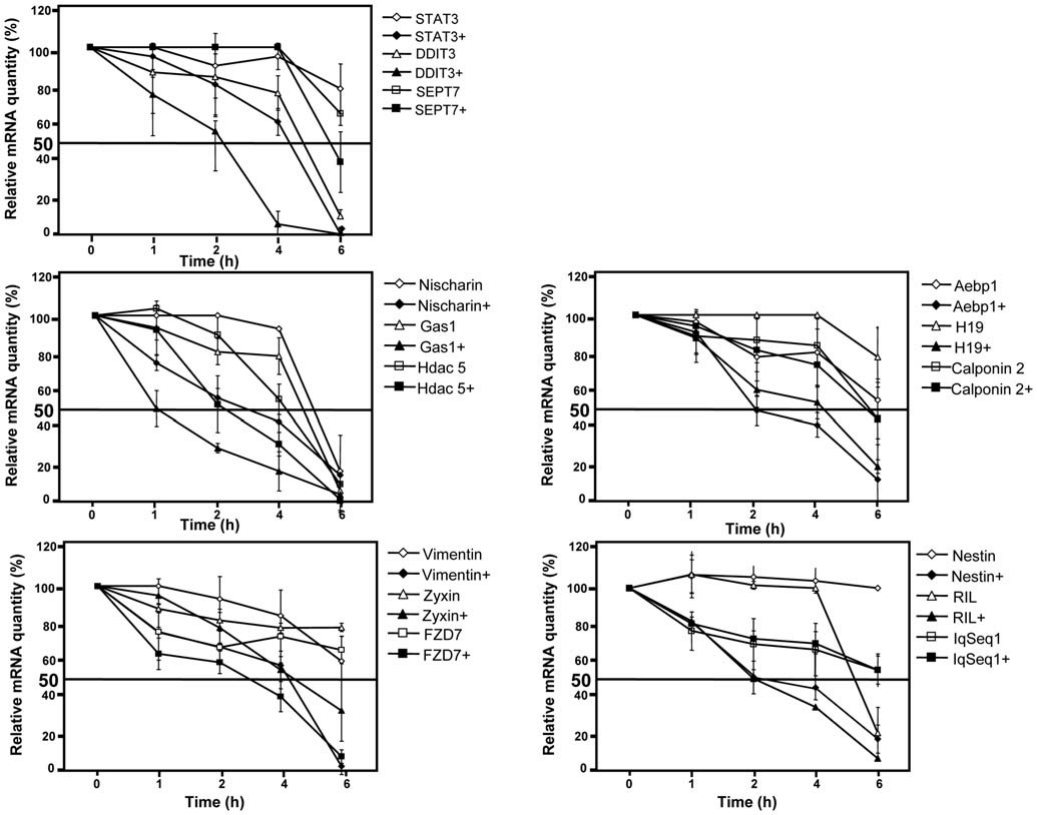
Measurement of mRNA stability. C2-RNase L cells were treated with 2 mM IPTG for 6 h and then Actinomycin D (5 µg/ml) was added to the cell culture medium. Cells were collected at the indicated time points and mRNAs were analyzed by RT-PCR amplification as in Figure 2. PCR products were then quantified with the Image J program. Error bars refer to the standard deviation obtained in three independent experiments.

As RNase L is an endoribonuclease, if these mRNAs were direct targets of RNase L, RNase L should regulate their expression at post-transcriptional level and decrease their half life. Thus, we quantified their levels in C2-RNase L cells after treatment, or not, with 2 mM IPTG and Actinomycin D to inhibit transcription (Figure 3 and Supplemental Table S3). Only the half life of Calponin 2, Septin 7 and IqSeq1 were not reduced following RNase L induction in comparison to control cells, suggesting that all the other transcripts could be directly regulated by RNase L. These results confirm that RNase L activation in C2C12 myogenic cells leads to selective mRNA degradation.

### Conditional over-expression of RNase L inhibits myogenesis and favors adipogenesis

Since our data indicate that RNase L regulates master genes that control pluripotency (Stat3, Fzd7), myogenesis (MyoD, Stat3, Hdac5) and adipogenesis (Chop-10/Ddit3, Aebp1), we decided to differentiate C2-RNase L cells after induction of RNase L during the multipotency phase. To this aim, C2-RNase L cells at 80% confluency were treated, or not, with 5 mM IPTG for 6 h and then placed in medium permissive for myotube differentiation (MDM).

**Figure 4:**
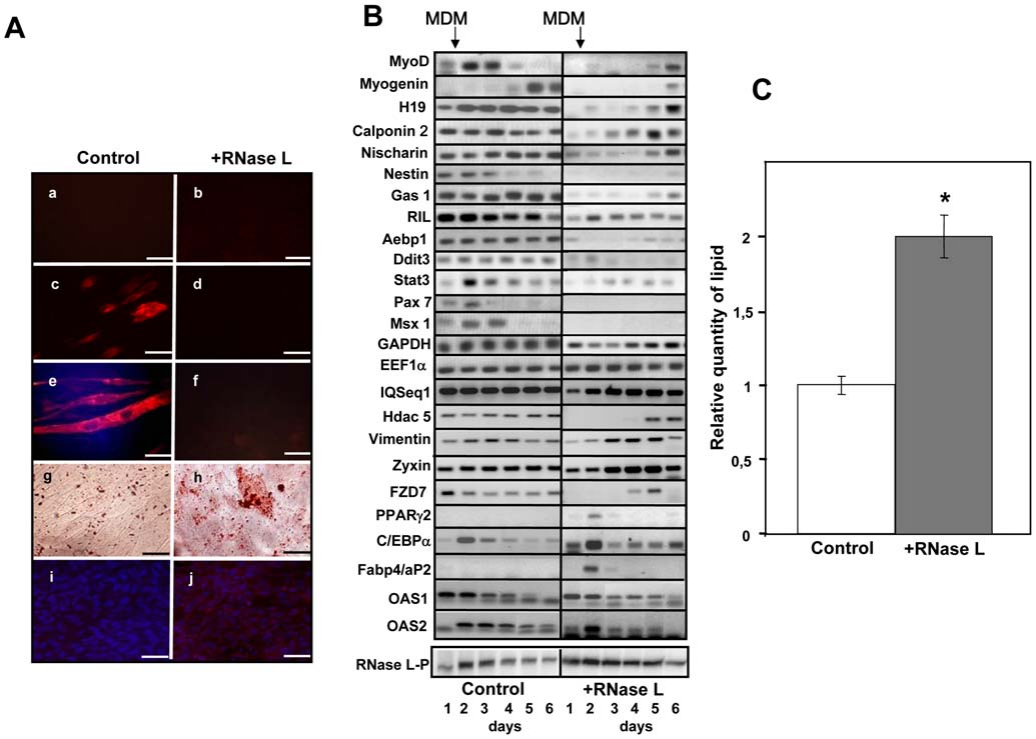
Induction of RNase L inhibits myotube differentiation and induces lipid accumulation. A: C2-RNase L cells were treated (+RNase L) or not (Control) with 5 mM IPTG for 6 h at day 0 (panels a and b), and then were induced to differentiate in MDM for 4 (panels c,d,i,j) or 6 days (panels e and f and panels g and h). At the different time points cells were fixed and incubated with a monoclonal antibody against Troponin-T (panels a to f) or stained with Oil-red-O (panels g and h). At day 4, cells were also analyzed with a monoclonal antibody against Fabp4/aP2 (panels i and j). Cells were observed at a magnification of 20x, (^__^): 60 µm. B: At day 1, C2-RNase L cells were treated (+RNase L) or not (Control) with 5 mM IPTG for 6 h and then were induced to differentiate in MDM. Cells were then collected at the indicated time points and expression of specific mRNAs was analyzed by RT-PCR amplification. PCR products were analyzed on 1.2% agarose gels. Photographs of the gels are shown. RNase L protein expression was analyzed as in Figure 1B. Autoradiographs of these gels are shown (RNase L-P). C: Quantification of lipids accumulated in C2-RNase L cells treated ( 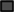 ) or not ( 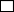 ) with 5 mM IPTG for 6 h and then induced to differentiate in MDM for 6 days. After staining with Oil-red-O, lipids were quantified by spectrophotometric analysis at 540 mm following elution of cell-retained Oil-red-O with isopropanol. The amount of lipids in untreated C2-RNase L cells was set to 1. Data were expressed as mean±SD of three different experimental points. (*) P<0.01 compared to non-induced cells.

Induction of RNase L expression clearly inhibited myotube differentiation (Figure 4A). Specifically, in C2-RNase L cells treated with IPTG, we did not observe cell fusion or expression of Troponin T, a protein only present in mature myotubes. Moreover, Myogenin began to be expressed later than in control cells and at lower level. Pax7 and Msx1, two genes involved in muscle differentiation, were also down-regulated (Figure 4B). These results indicate that conditional expression of RNase L inhibits muscle differentiation of C2C12 myogenic cells.

We then compared the expression of the genes that have been identified in this work as down-regulated by RNase L during muscle differentiation in control and IPTG-treated cells. Upon induction of RNase L, we observed a decrease in the expression of key regulators of cell fate (Stat3, Aebp1, Chop10/Ddit3, Hdac5, Fzd7) and also of genes which control cytoskeleton organization, cell shape, differentiation and signal transduction (Gas1, Nestin, Nischarin, Zyxin, Vimentin, Calponin 2) (Figure 4B). It must be noted that down-regulation was observed at day 1 after RNase L induction, but expression of the different mRNAs was generally restored at day 5–6 concomitantly with the decrease of RNase L activity (see Figure 1A) and expression (Figure 4B).

Since induction of RNase L led to concomitant down-regulation of MyoD, which induces muscle differentiation, and of Aebp1 and Chop10/Ddit3, which repress adipocyte differentiation [51], [59], we asked whether in the cells treated with IPTG, adipogenesis would be favored whilst myogenesis was inhibited. Indeed, staining with Oil red-O, a specific dye for lipids, showed a significant accumulation of lipids in C2-RNase L cells treated with IPTG compared to untreated cells, despite being grown in MDM (Figure 4A, panels g and h). Quantification of lipids by spectrophotometric analysis at 540 mm after elution of cell-retained Oil-red-O with isopropanol [25] indicated that C2-RNase L cells accumulated two times more lipids than untreated cells (Figure 4C). Adipogenic differentiation is under the control of several genes, such as the CCAAT/enhancer binding protein-α (C/EBPα) [60] and peroxisome proliferator activator receptor gamma 2 (PPARγ2) [61], and it is characterized by expression of some specific proteins like the fatty acid binding protein 4/adipocyte protein 2 (Fabp4/aP2) [62]. We therefore quantified their expression in C2-RNase L cells grown in MDM after treatment, or not, with 5 mM IPTG for 6 h (Figure 4B). C/EBPα was expressed both in control and RNase L-induced cells but at different levels; whereas PPARγ2 and Fabp4/aP2 were transiently induced only upon treatment with IPTG (Figure 4B). Expression of Fabp4/aP2 was also observed at the protein level (Figure 4A, panels i and j). Moreover, we could confirm that 2-5A-synthetase 1 (OAS1) and 2-5A-synthetase 2 (OAS2) were induced during differentiation (Figure 4B). These findings indicate that, upon transient induction of RNase L, lipid accumulation was increased in cells cultured in conditions permissive for muscle differentiation. However, RNase L activation was not sufficient by itself to induce complete adipocyte differentiation in MDM, a non permissive medium for adipocyte differentiation. In addition, treatment of C2-RNase L cells with 5 mM IPTG for 6 h every two days after induction of muscle differentiation also did not lead to complete adipocyte differentiation and induced massive cell death (Supplemental Figure S1). In fact, in previous works carried out in several cell models it was also reported that over-activation of RNase L stimulated apoptosis [34], [35].

**Figure 5:**
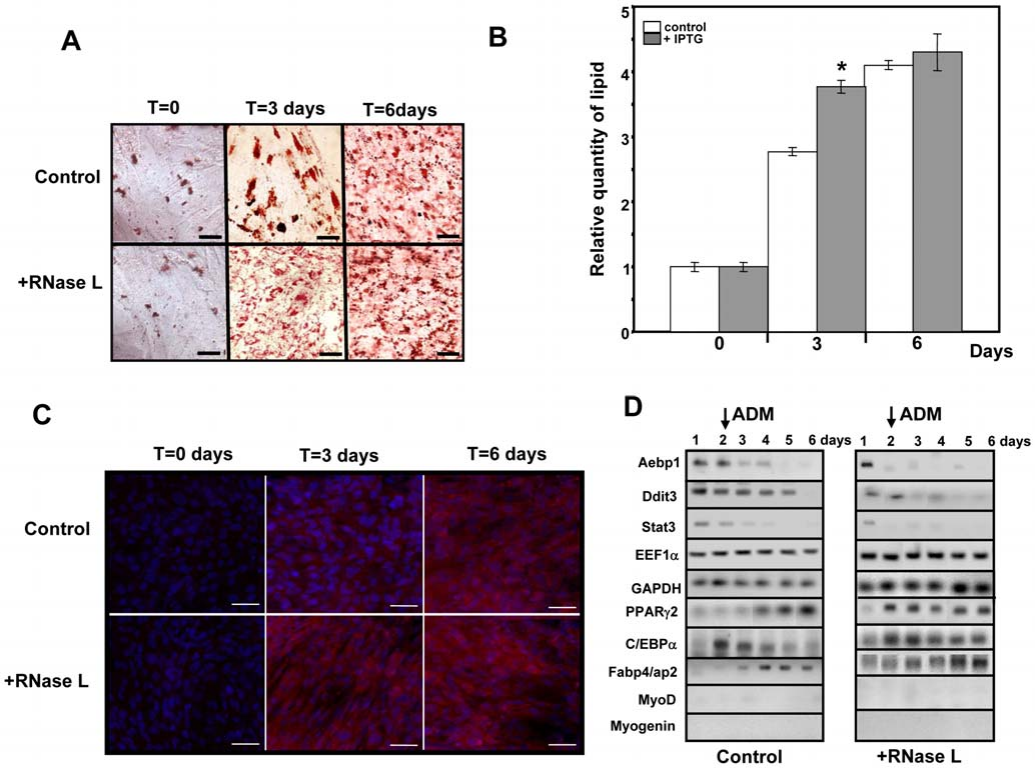
Increased RNase L levels favor adipocyte differentiation. A: C2-RNase L cells were treated (+RNase L) or not (Control) with 5 mM IPTG for 6 h at day 0 and then induced to differentiate in ADM. Cells were fixed and stained with Oil-red-O to reveal lipids accumulation at day 0, 3 and 6. Cells were observed at a magnification of 20x, (^__^): 60 µm. B: Quantification of lipids in control (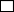) or IPTG-treated C2-RNase L cells ( 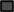 ) at day 0, 3 and 6 after induction of differentiation in ADM. After staining with Oil-red-O, lipids were quantified by spectrophotometric analysis at 540 mm following elution of cell-retained Oil-red-O with isopropanol. The amount of lipids in C2-RNase L cells at T = 0, before addition of IPTG, was set to 1. Error bars refer to the standard deviation obtained in three independent experiments. (*) P<0.01 compared to untreated cells at T = 3. C: C2-RNase L cells were treated (+RNase L) or not (Control) with 5 mM IPTG for 6 h at day 0 and then induced to differentiate in ADM. Cells were fixed at day 0, 3 and 6 and then incubated with an antibody against Fabp4/aP2 (Red). DNA was stained with Dapi (Blue). Cells were observed at 20x, (^__^): 50 µm. D: Analysis of mRNA expression by RT-PCR amplification at different time points during adipocyte differentiation. RNase L was induced (+RNase L) or not (Control) with 5 mM IPTG for 6 h at day 2 and then cells were switched to ADM to induce adipocyte differentiation. PCR products were analyzed by gel electrophoresis. Photographs of the gels are shown.

We then followed adipocyte differentiation by measuring lipid accumulation (Figure 5A and 5B), Fabp4/aP2 expression (Figure 5C), Perilipin expression (Supplemental Figure S3) and gene expression (Figure 5D) in control and C2-RNase L cells treated with IPTG in medium permissive for adipocytes differentiation (ADM). It must be noted that control C2-RNase L cells displayed a strong adipogenic differentiation even in the absence of PPAR γ activator (Figure 5A and B). Therefore, we compared adipogenic differentiation in control C2-RNase L cells and parent C2C12 cells grown in ADM (Supplemental Figure S2). Quantification of lipids after 6 days of differentiation showed that control C2-RNase L cells accumulated 25% more lipids than parent C2C12 cells. This difference could be due the slight increase in RNase L level observed during the multipotency period in control C2-RNase L cells in comparison to parent C2C12 cells even without induction with IPTG (Figure S2, panel C). This difference could be caused by a leak in the repressor system of the C2-RNase L cells.

Nevertheless, adipocyte differentiation was accelerated by RNase L induction: lipid accumulation and Fabp4/aP2 expression was significantly increased at day 3 of differentiation in RNase L-induced cells in comparison to control cells (Figure 5A, B and C). Again, we could observe down-regulation of genes which play a key role in cell fate commitment, such as Aebp1 and Chop-10/Ddit3 (Figure 5D). PPARγ2 and Fabp4/aP2 mRNA were expressed early in RNase L-induced cells in comparison to control cells (Figure 5D).

**Figure 6:**
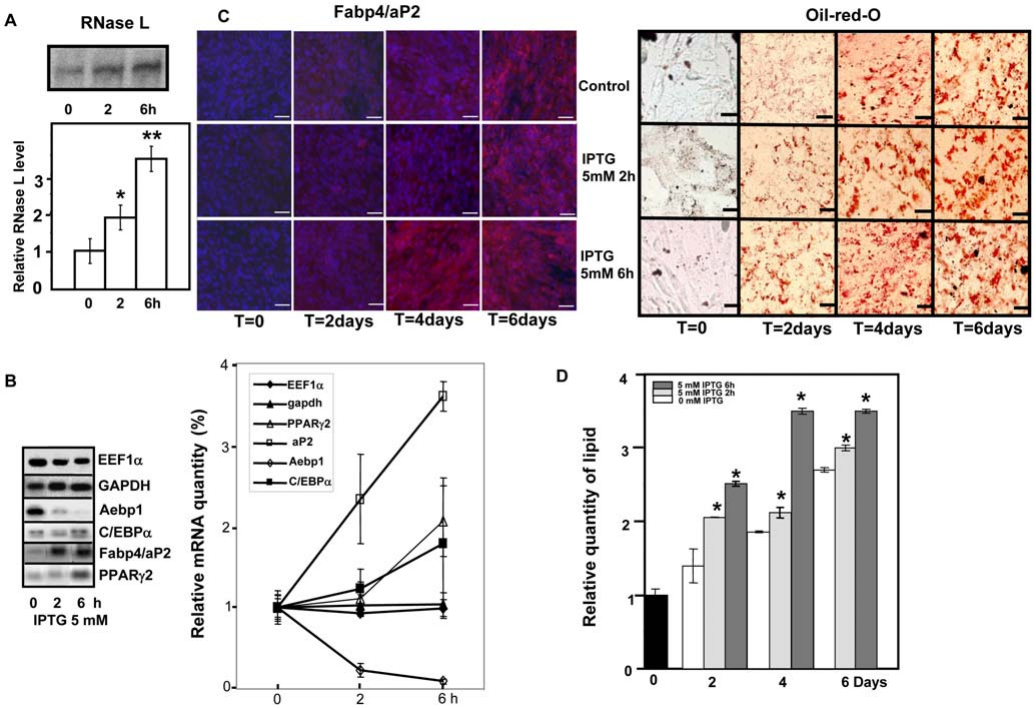
The kinetics of adipocyte differentiation depends on the RNase L level. A: C2-RNase L cells were treated, or not (0), with 5 mM IPTG for (2) or (6)h. Cells were then harvested and analyzed for RNase L 2-5A binding activity with the 2-5A radio-covalent binding assay. Proteins were separated on 10% polyacrylamide gels. A representative autoradiography of the gel and a densitometry of the gel are shown. The amount of 2-5A binding activity in C2-RNase L cells in the absence of IPTG was set to 1. Error bars refer to the standard deviation obtained in three independent experiments. (*) P<0.0 compared to non- induced cells (**) P<0.01 compared to control, untreated cells. B: RNase L was induced by 5 mM IPTG for 0, 2 or 6 hours, and then mRNA expression was analyzed by RT-PCR amplification and agarose gel electrophoresis. Photographs and densitometric analysis of the gels are shown. The level of the different mRNAs in untreated cells was set at 1. Quantification of EEF1α mRNA was used as a control of semi-quantitative RT-PCR experiment. Error bars refer to the standard deviation obtained in three independent experiments. C: C2-RNase L cells were treated, or not, with 5 mM IPTG for 2 or 6 h and then induced to differentiate in ADM. At T = 0, T = 2, T = 4 and T = 6 days after induction of differentiation, cells were fixed and stained with Oil-red-O to reveal lipid accumulation (panel on the right) or incubated with an antibody against Fabp4/aP2 (Red) (panel on the left); DNA was stained with Dapi (Blue). Cells were observed at 20x, (^__^): 50 µm. D: Quantification of lipids in control or IPTG-treated C2-RNase L cells (for 2 or 6 h) at T = 0, T = 2, T = 4 and T = 6 days after induction of differentiation with ADM. After staining with Oil-red-O, lipids were quantified by spectrophotometric analysis at 540 mm following elution of cell-retained Oil-red-O with isopropanol. The amount of lipids in C2-RNase L cells at T = 0, before IPTG treatment, was set to 1. Error bars refer to the standard deviation obtained in three independent experimental points. (*) P<0.01 compared to untreated cells at the same time during adipocyte differentiation.

Since a 6 h treatment with IPTG accelerated adipocyte differentiation, we asked whether a shorter treatment could be sufficient. To this aim we incubated C2-RNase L cells with 5 mM IPTG for different lengths of time. A 6 h treatment produced a slightly higher concentration of RNase L protein (Figure 6A) and a faster adipocyte differentiation than in control cells or cells treated with IPTG for only 2 hours as shown by C/EBPα, PPARγ2, Fabp4/aP2 mRNA expression and Aebp1 mRNA decrease (Figure 6B). These results were confirmed also by histological analysis of Fabp4/aP2 expression (Figure 6C) and quantification of lipid accumulation (Figure 6D).

### Inhibition of RNase L impairs adipocyte differentiation and favors myocyte differentiation

**Figure 7:**
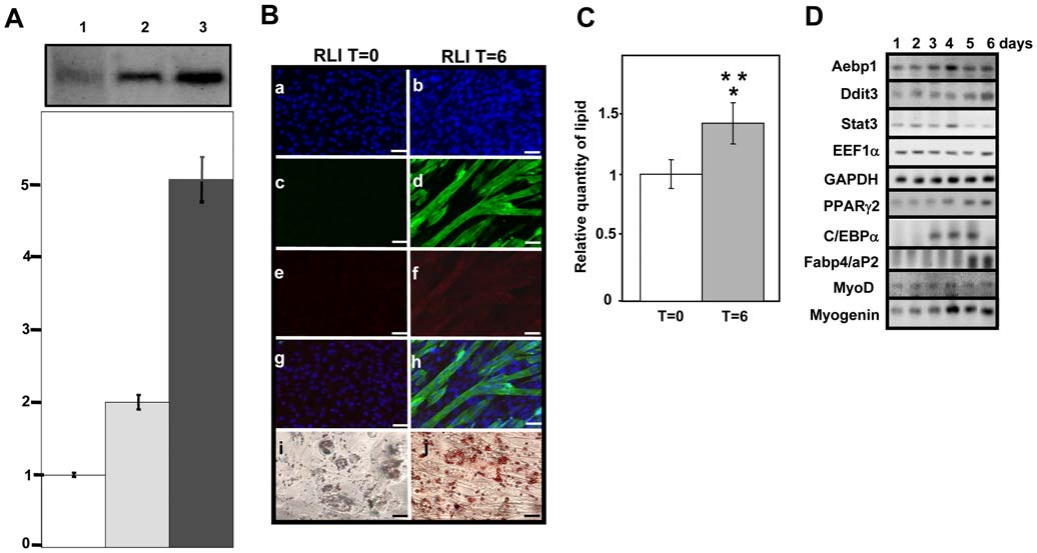
Inhibition of adipocyte differentiation by RLI. A: RNase L activity in C2-RNase L cells, C2-RNase L cells treated with IPTG and C2-RLI cells. C2-RLI cells (1,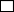), C2-RNase L cells (2,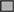 ) and C2-RNase L treated with 5 mM IPTG (3,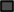 ) for 6 h were analyzed for RNase L 2-5A binding activity with the 2-5A radiocovalent binding assay. Proteins were separated in 10% polyacrylamide gels. A representative autoradiography and a densitometry of the gel are shown. The amount of 2-5A binding activity in C2-RLI cells was set to 1. Error bars refer to the standard deviation obtained in three independent experiments. B: C2-RLI differentiation. Cells were fixed and incubated with Oil-red-O to reveal lipid accumulation (i = day 0, j = day 6 after induction of differentiation with ADM) and expression of Troponin T and Fabp4/aP2 was analyzed using monoclonal antibodies against Troponin T (Green, c,d) and Fabp4/aP2 (Red e,f) at day 0 (a,c,e,g,i) and day 6 after induction of differentiation with ADM (b,d,f,h,j). DNA was stained with Dapi (Blue a,b,g,h). A merge of Dapi, Troponin T and Fabp4/aP2 labeling is shown (g,h). Cells were observed at 20x, (^__^): 20 µm. C: Quantification of lipids in C2-RLI cells at day 0 (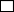) and day 6 after induction of differentiation with ADM ( 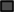 ). A value of 1 corresponds to the amount of lipids in C2-RLI cells at T = 0, before differentiation. After staining with Oil-red-O, lipids were quantified by spectrophotometric analysis at 540 mm following elution of cell-retained Oil-red-O with isopropanol. Error bars refer to the standard deviation obtained in three independent experiments. (*) P<0.01 compared to untreated C2-RNase L cells and (**) P<0.01 compared to IPTG-treated C2-RNase L cells at the same time point during adipocyte differentiation. D: Analysis of mRNA expression by RT-PCR amplification at different time points during adipocyte differentiation. C2-RLI cells were switched to ADM to induce adipocyte differentiation at day 1. PCR products were analyzed by gel electrophoresis. Photographs of the gels are shown.

RNase L activity can be regulated by interaction with RLI which inhibits its activation by 2-5A [63]. We previously determined that RLI over-expression in C2C12 cells (C2-RLI cells) accelerates myotube formation [20] and that in C2-RLI cells RNase L activity is lower than in control C2C12 cells [20]. Here we show that, in C2-RLI cells, RNase L activity is 50% lower than in C2-RNase L cells and 80% lower than in C2-RNase L cells treated with IPTG (Figure 7A). We thus asked whether inhibition of RNase L activity by RLI could influence the choice of differentiation pathway of C2C12 cells. To this aim we switched C2-RLI cells to ADM and followed their differentiation. C2-RLI cells accumulated about 60% fewer lipids than C2-RNase L cells treated with IPTG (compare Figures 7B, panels i and j, and 7C with Figures 5B and 6D) and even less than untreated C2-RNase L cells. Expression of Fabp4/aP2 and of Perilipin, another adipogenic marker, was low (Figure 7B, panels e and f and Supplemental Figure S3). Moreover, although C2-RLI cells were cultured in ADM, we observed formation of myotubes which expressed Troponin T, a protein specific of mature myotubes (Figure 7B, panels d and h and Supplemental Figure S3). In these cells, MyoD and Myogenin were expressed concomitantly with aP2 and PPARγ2 (Figure 7D). In addition, Aebp1 and Chop-10/Ddit3 were not down-regulated after induction of adipocyte differentiation (Figure 7D).

## Discussion

Here we report that RNase L and its inhibitor RLI play a central role in determining muscle cell fate. Myogenesis is controlled by several functionally coherent mechanisms that are precisely coordinated and regulated in time to allow the sequence of activation/inactivation of genes expression. Muscular differentiation is the result of the interplay between several mechanisms such as transcriptional induction, transcriptional repression and mRNA stability. mRNA stability is now recognized as an essential mechanism of control of gene expression [64] and here we show that its regulation via RNase L is important for muscle differentiation.

RNase L and RLI are sequentially induced during muscular differentiation [15], [20], [22], [65] and Figures 1 and 4. Since RNase L is expressed in a restricted window of time during muscle differentiation, it could play an essential role in regulating the stability of mRNAs encoding for factors which need to be precisely monitored during myogenesis. Indeed, we previously showed that RNase L regulates MyoD mRNA stability [20] and in this work we identified by SAGE analysis 166 mRNAs which are down-regulated upon induction of RNase L during the multipotency phase, when C2C12 cells are still proliferating and can differentiate into muscle or adipose tissues (Table S1 and Figures 1 and 2). Specifically, several factors controlling muscle stem cell proliferation, cell fate and differentiation, such as Fzd7, Stat3, IqSec1, Aebp1, Ddit3, Hdac5, H19, were down-regulated as well as several transcripts implicated in the structure of actin cytoskeleton (Nischarin), stress fibers (RIL, Septin 7), focal adhesion (Zyxin), intermediate filaments (Nestin, Vimentin) or in Ca2+ signaling (Calponin 2). Stress fibers and focal adhesion play a central role in actin cytoskeleton remodeling during muscle differentiation. Membrane-cytoskeleton interaction regulates transmembrane currents and Ca2+ influx and participates in signal transduction leading to the regulation of numerous cellular processes such as gene expression and cell differentiation and in particular cell fusion [66], [67].

Although RNase L has no great sequence specificity [68], its activation influenced the stability of only a small numbers of mRNAs (Table S1). Similarly, a limited number of transcripts was identified following 2-5A transfection of prostate cancer cells [69]. The stability of some mRNAs is determined by AU-rich elements [70], [71]. However, it was not the case for the mRNA identified in this study. We could not find common nucleic acid motifs among the identified mRNAs.

Such specificity could be due to interaction of RNase L with trans-acting factors involved in its recruitment to target RNAs, such as eRF3 (eukaryotic Release Factor 3) a factor implicated in mRNA translation [72] and IF2mt (mitochondrial Initiation Factor 2) a factor implicated in mitochondrial mRNA translation [35]. In fact, the mRNAs we identified in this work encode factors expressed during the proliferative and multipotency periods of myogenic cells. These mRNA are translated when RNase L is activated. We have previously shown that activation of RNase L by 2-5A induced its interaction with eRF3 and consequently its association with the mRNA translation complex [72]. Moreover, it has been shown that RNase L regulates mRNA stability during their translation [35], [72].

Our results indicate that after RNase L induction muscle cell fate is modified. RNase L seems to be implicated in the transition between proliferation and differentiation of myogenic cells. However, its premature induction led to down-regulation of MyoD and also of Aebp1 and Chop-10/Ddit3 which inhibit adipogenesis [51], [53]. Normally, their expression is stable during myogenesis, whereas in IPTG-treated C2-RNase L cells they were no longer expressed even when cells were induced to differentiate into muscles. As a consequence, myogenesis was inhibited in favor of adipogenesis as RNase L-induced cells accumulated more lipids than control cells and transiently expressed PPARγ2 and Fabp4/aP2 (Figures 4, 5 and 6). However, transient RNase L activation was not sufficient by itself to induce complete adipocyte differentiation in MDM, a non permissive medium for adipocyte differentiation. On the other way, in ADM, RNase L induction accelerated adipocyte differentiation (Figures 5 and 6), but did not drastically modify the pattern of gene expression during adipocyte differentiation as observed in MDM.

As conditional expression of RNase L inhibits myogenesis and seems to favor adipocyte differentiation, we then inhibited RNase L by over-expressing RLI, the RNase L inhibitor [63]. Inhibition of RNase L by RLI prevented down-regulation of Aebp1 and Chop-10/Ddit3 as well as of MyoD and, as a consequence, C2C12 cells differentiated into myotubes although they were grown in a culture medium permissive for adipocyte differentiation. Regulated expression of MyoD [73], Aebp1 [59], PPARγ and Chop-10/Ddit3 [74], [75] is crucial for the balance between myoblast and adipocyte differentiation. Here we show that deregulated expression of RNase L, an enzyme that controls mRNA stability of these key factors, can have negative effects on myogenesis.

Our results show that RNase L seems to be implicated in the transition between proliferation and differentiation of myogenic stem cells. Modification in the kinetics of RNase L activation and inhibition leads to important perturbations in the choice of differentiation pathway (myogenesis versus adipogenesis) of C2C12 myogenic cells. Myogenesis is required not only for normal muscle development, but also for maintenance and repair of adult myofibers. Impairment in the process of muscle regeneration has been proposed as one of the important determinants of skeletal muscle wasting especially in aging and chronic diseases such as Duchenne muscular dystrophy (DMD). In these conditions, accumulation of adipose tissue is observed in skeletal muscle [5], [76]. Adipose tissue development is associated with degenerative/regenerative or atrophic changes in skeletal muscle fibers and a common feature of these pathologies is a chronic inflammatory state of muscles. During chronic inflammation, several cytokines are produced continuously not only by immune cell infiltrates but also by muscle cells [77], [78]. Among these different cytokines IFN is an important regulator of RNase L [79]-[82]. If the role of IFN and inflammation has been widely studied and begins to be understood in human muscle regeneration, the role of enzymatic pathways regulated by IFN is still unknown. In particular, the role of RNase L has not yet been established in primary myoblasts. Our results show that the level of RNase L activity could modify muscle cell differentiation and engage them into adipocyte differentiation. On the other way, owing to their capacity to regenerate damaged muscle, satellite cells have been considered as candidates for cell based therapies to treat muscular dystrophies or other muscular diseases. Understanding the role of RNase L/RLI in the control of proliferation and induction of alternative differentiation pathways in satellite cells during inflammation is of medical importance and is central for the development of therapies based on their use.

## Supporting Information

Figure S1 C2-RNase L cells were treated with 5 mM IPTG for 6 h at day 0 and then: i) they were induced to differentiate in MDM (panels a); or ii) they were induced to differentiate in MDM and were treated again with 5 mM IPTG for 6 h at day 2 (panels b); or iii) they were induced to differentiate in MDM and were treated again with 5 mM IPTG for 6 h at day 2 and day 4 (panels c). At day 6, all the cells were fixed and, stained with Oil-red-O (panels a, b and c). Cells were observed at 20x, (__): 50 Âµm.
(1.17 MB TIF)

Figure S2 A: C2C12 and C2-RNase L cells were plated at high density in GM and shifted to ADM at confluence. At day 6, cells were fixed and stained with Oil-red-O. Cells were observed at 20x, (__): 50 Âµm. B: Quantification of lipids in differentiated C2C12 and C2-RNase L cells at day 6 after induction of differentiation with ADM. A value of 1 corresponds to the amount of lipids in C2C12 cells at day 6. Error bars refer to the standard deviation obtained in three independent experimental points. C: RNase L binding to 2-5A. C2C12 and C2-RNase L cells were plated at high density in GM (day 1). At day 3 (i.e., 80% confluence, multipotency period), cells were harvested and analyzed for RNase L binding to 2-5A with the 2-5A radiocovalent binding assay. Proteins were separated on 10% polyacrylamide gels. A representative autoradiography and a densitometry of the gel are shown.
(0.83 MB TIF)

Figure S3 C2-RNase L cells (a), C2-RNase L cells treated with IPTG 5 mM for 6 h (b) and C2-RLI cells were plated at high density in GM and shifted to ADM at confluence. At day 6, cells were fixed and expression of Perilipin (red) and Troponin T (green) was analyzed using antibodies. DNA was stained with Dapi (Blue). A merge of Dapi, Troponin T and Perilipin labeling is shown. Cells were observed at 20x, (__): 20 µm.
(3.50 MB TIF)

Table S1 Down-regulated genes. List of the 166 genes that were down-regulated more than three folds in C2-RNase L cells after RNase L induction and their level in control C2-RNase L cells.
(0.16 MB XLS)

Table S2 PCR primers used to study the expression of the different transcripts
(0.02 MB XLS)

Table S3 Half life of mRNAs down-regulated upon RNase L induction. Half lives were calculated by non linear regression analysis of the percentage of mRNA remaining as a function of time after Actinomycin D treatment of C2-RNase L cells incubated or not with 2 mM IPTG for 6 hours. Data are the mean of three independent experiments.
(0.04 MB DOC)
