# Supplementary figures and images for: Endoribonuclease L (RNase L) Regulates the Myogenic and Adipogenic Potential of Myogenic Cells
Source: PLoS One. 2009 Oct 23;4(10):e7563. doi: 10.1371/journal.pone.0007563 (PMC2762314; doi:10.1371/journal.pone.0007563)

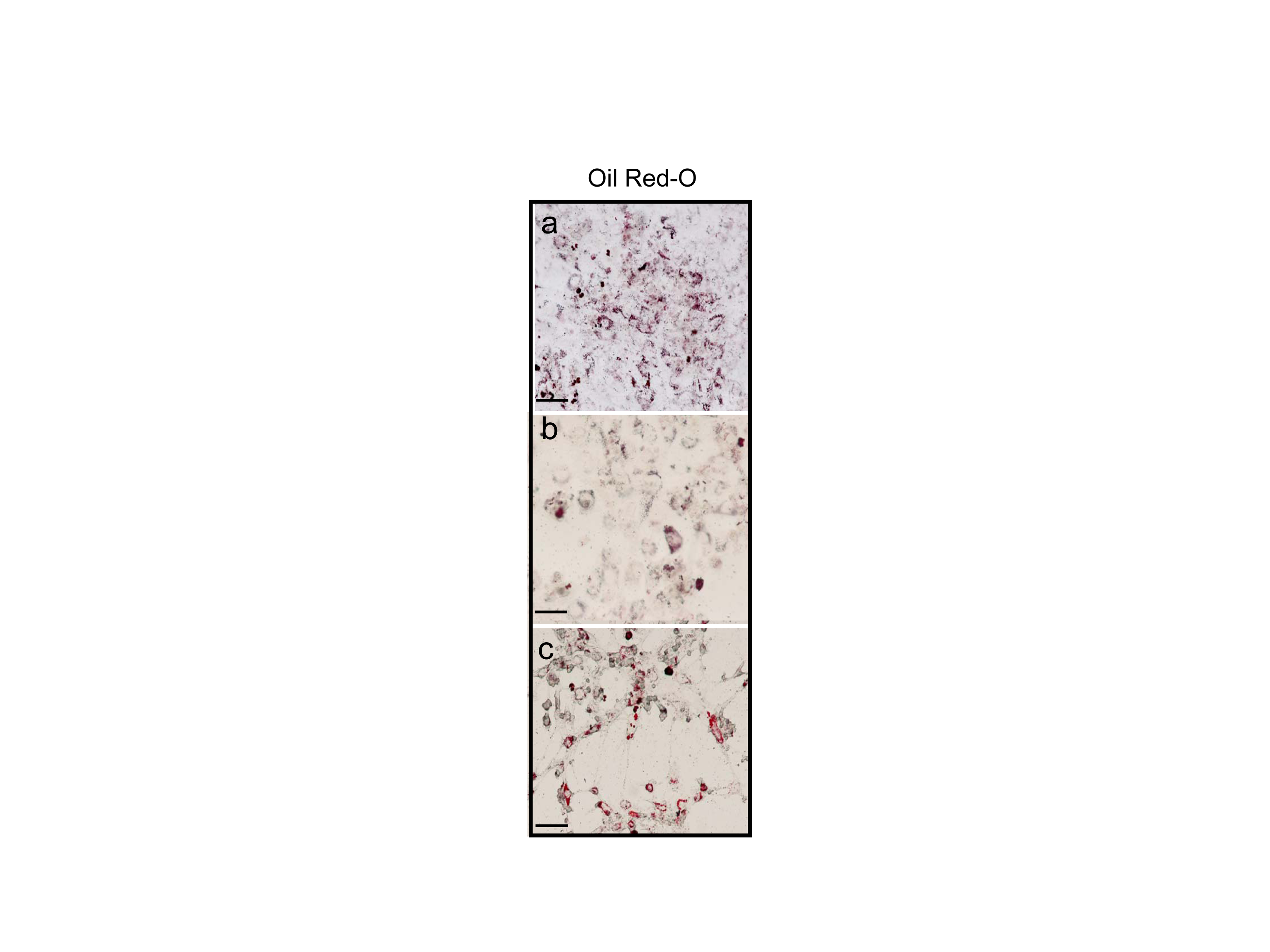

Supplement: Figure S1 — C2-RNase L cells were treated with 5 mM IPTG for 6 h at day 0 and then: i) they were induced to differentiate in MDM (panels a); or ii) they were induced to differentiate in MDM and were treated again with 5 mM IPTG for 6 h at day 2 (panels b); or iii) they were induced to differentiate in MDM and were treated again with 5 mM IPTG for 6 h at day 2 and day 4 (panels c). At day 6, all the cells were fixed and, stained with Oil-red-O (panels a, b and c). Cells were observed at 20x, (__): 50 Âµm. (1.17 MB TIF) [file pone.0007563.s001.tif]

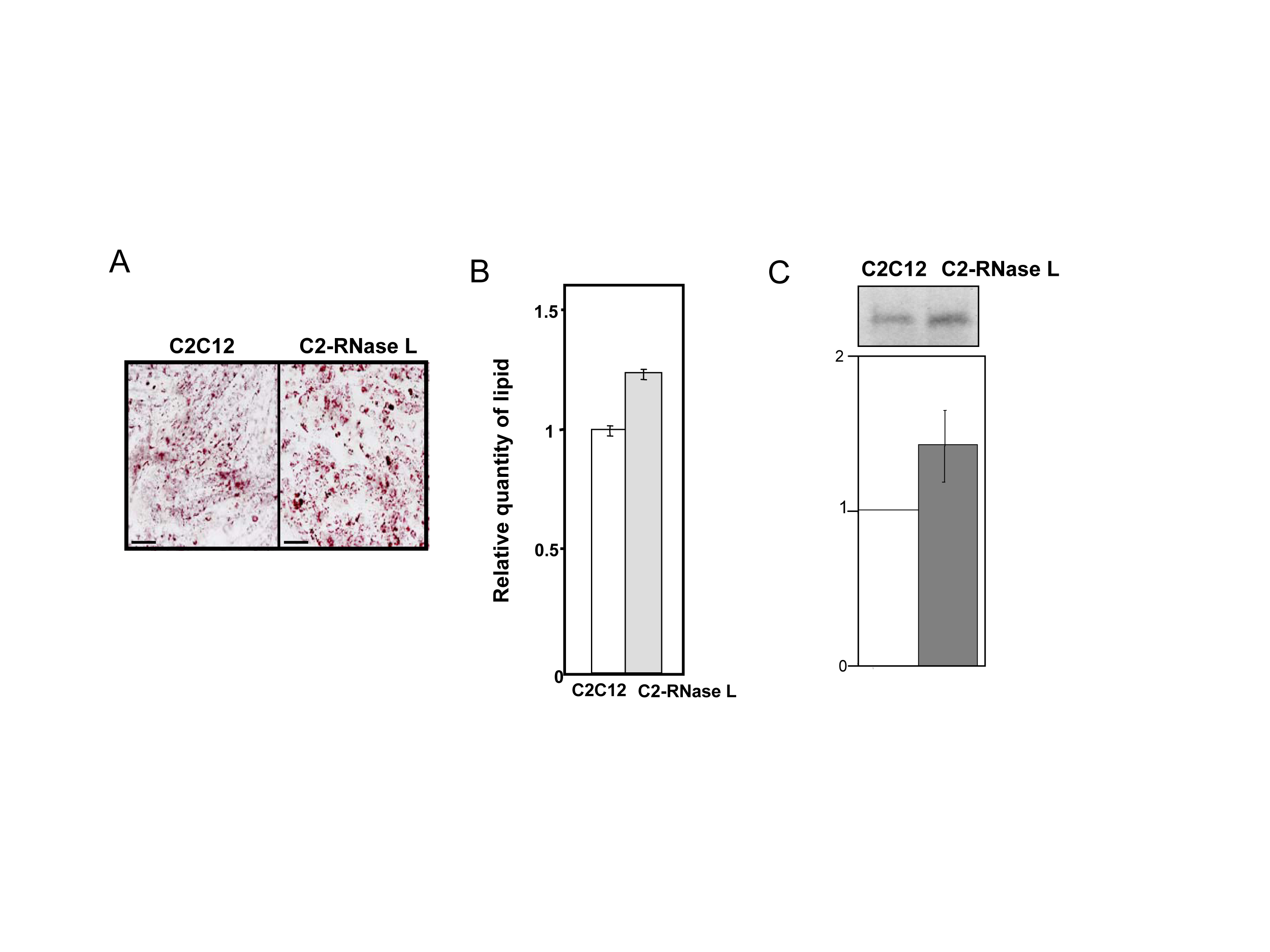

Supplement: Figure S2 — A: C2C12 and C2-RNase L cells were plated at high density in GM and shifted to ADM at confluence. At day 6, cells were fixed and stained with Oil-red-O. Cells were observed at 20x, (__): 50 Âµm. B: Quantification of lipids in differentiated C2C12 and C2-RNase L cells at day 6 after induction of differentiation with ADM. A value of 1 corresponds to the amount of lipids in C2C12 cells at day 6. Error bars refer to the standard deviation obtained in three independent experimental points. C: RNase L binding to 2-5A. C2C12 and C2-RNase L cells were plated at high density in GM (day 1). At day 3 (i.e., 80% confluence, multipotency period), cells were harvested and analyzed for RNase L binding to 2-5A with the 2-5A radiocovalent binding assay. Proteins were separated on 10% polyacrylamide gels. A representative autoradiography and a densitometry of the gel are shown. (0.83 MB TIF) [file pone.0007563.s002.tif]

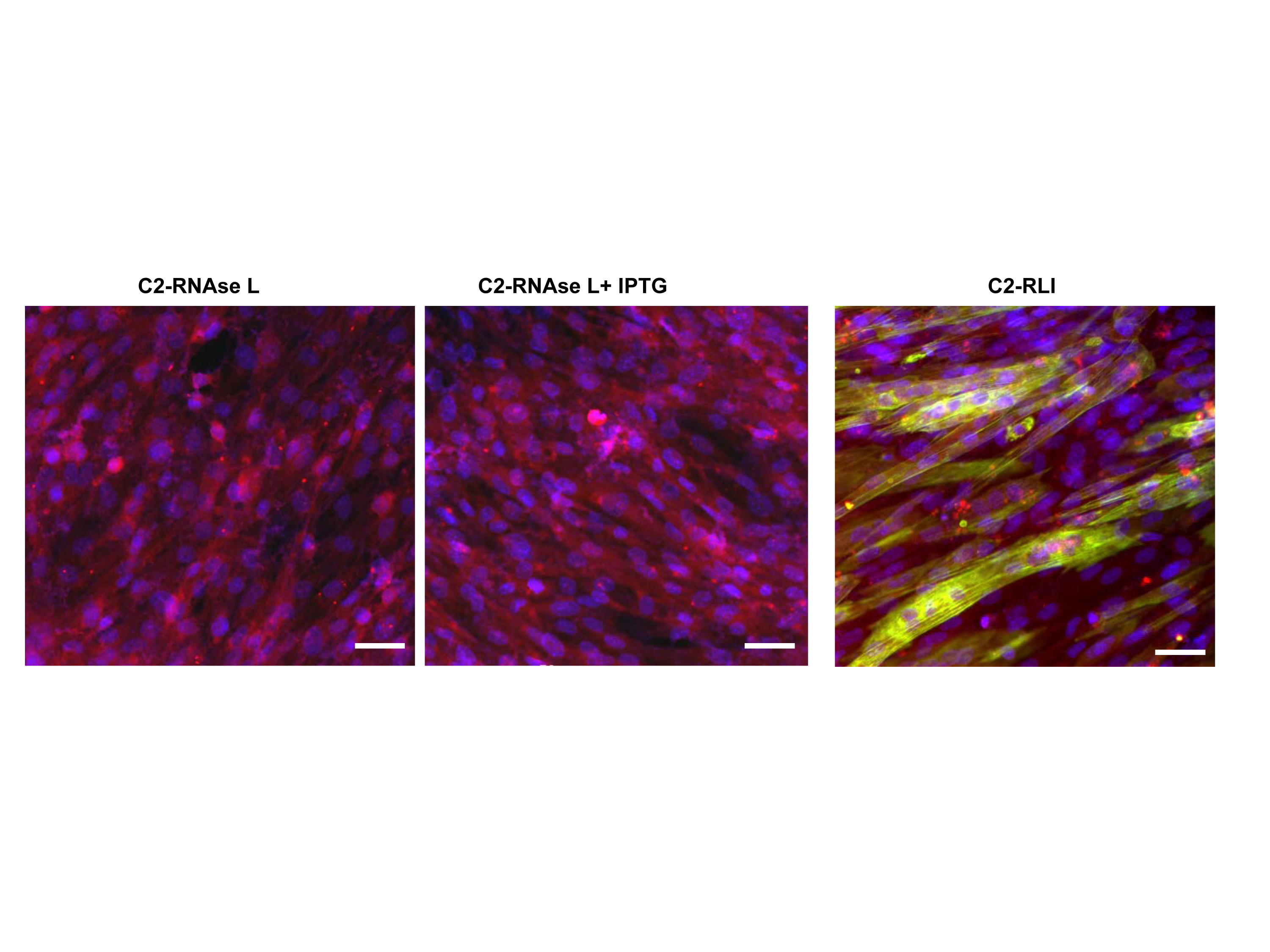

Supplement: Figure S3 — C2-RNase L cells (a), C2-RNase L cells treated with IPTG 5 mM for 6 h (b) and C2-RLI cells were plated at high density in GM and shifted to ADM at confluence. At day 6, cells were fixed and expression of Perilipin (red) and Troponin T (green) was analyzed using antibodies. DNA was stained with Dapi (Blue). A merge of Dapi, Troponin T and Perilipin labeling is shown. Cells were observed at 20x, (__): 20 µm. (3.50 MB TIF) [file pone.0007563.s003.tif]
