# Supplementary material for: Endoribonuclease L (RNase L) Regulates the Myogenic and Adipogenic Potential of Myogenic Cells
Source: PLoS One. 2009 Oct 23;4(10):e7563. doi: 10.1371/journal.pone.0007563 (PMC2762314; doi:10.1371/journal.pone.0007563)
Supplement: Table S3 — Half life of mRNAs down-regulated upon RNase L induction. Half lives were calculated by non linear regression analysis of the percentage of mRNA remaining as a function of time after Actinomycin D treatment of C2-RNase L cells incubated or not with 2 mM IPTG for 6 hours. Data are the mean of three independent experiments. (0.04 MB DOC) [file pone.0007563.s006.doc]

**Table S3 : mRNA half lives.**

**3h10**

**>6h**

**FZD7**

**4h20**

**>6h**

**Zyxin**

**4h20**

**>6h**

**Vimentin**

**4h**

**4h**

**IQSeq1**

**2h**

**5h10**

**RIL**

**2h**

**>6h**

**Nestin**

**2h**

**4h20**

**Hdac 5**

**1h**

**5h**

**Gas 1**

**2h**

**5h30**

**Nischarin**

**6h**

**6h**

**Calponin 2**

**4h35**

**> 6h**

**H19**

**4h**

**>6h**

**Aebp1**

**5h40**

**> 6h**

**Septin 7**

**2h15**

**4h50**

**Ddit3**

**4h20**

**> 6h**

**Stat 3**

**RNase L**

**Control**
